# Comparative Evaluation of Osteoarthritic Changes in the Temporomandibular Joint Using Cone-Beam Computed Tomography Among Individuals With and Without Temporomandibular Disorders: A Comprehensive Review

**DOI:** 10.7759/cureus.115183

**Published:** 2026-08-25

**Authors:** Prajakta C Khelkar, Ayushi P Mangulkar, Chetan Fukate, Nilam Sonawane, Snehal Chavhan, Kunal Fule, Freny R Karjodkar

**Affiliations:** 1 Department of Dentistry, Government Medical College and Hospital, Bhandara, IND; 2 Department of Dentistry, Government Medical College and Hospital, Sambhaji Nagar, IND; 3 Department of Dentistry, Government Medical College and Hospital, Amravati, IND; 4 Department of Dentistry, BJ Government Medical College and Hospital, Pune, IND; 5 Department of Dentistry, Government Medical College and Hospital, Nandurbar, IND; 6 Oral Medicine and Radiology, Karjodkar’s Dental Clinic, Mumbai, IND

**Keywords:** condylar erosion, cone-beam computed tomography, degenerative joint disease, mandibular condyle, osteoarthritis, osteophyte, temporomandibular disorders, temporomandibular joint

## Abstract

Osteoarthritis (OA) of the temporomandibular joint (TMJ) is a common degenerative condition and radiographic finding among individuals evaluated for temporomandibular disorders (TMDs), characterized by osseous changes such as flattening, subchondral sclerosis, erosion, osteophyte formation, and subchondral cysts. Cone-beam computed tomography (CBCT) provides three-dimensional visualization of TMJ osseous structures without superimposition and is particularly valuable for detecting degenerative changes, although its ability to assess soft-tissue structures is limited. This narrative review summarizes current evidence on the prevalence and distribution of CBCT-detected TMJ osseous changes in TMD and non-TMD populations and evaluates the influence of demographic variables, including age and sex, on these findings. Across the reviewed literature, osseous changes were generally more prevalent and severe among individuals with TMD than among those without TMD, although substantial overlap was observed between groups, indicating that imaging findings should be interpreted alongside validated clinical diagnostic criteria rather than used as a stand-alone diagnostic tool. Emerging approaches, including three-dimensional superimposition, low-dose acquisition protocols, quantitative assessment, and artificial intelligence-assisted interpretation, may improve the consistency and clinical utility of CBCT-based evaluation of TMJ osseous changes.

## Introduction and background

The temporomandibular joint (TMJ) is a synovial, ginglymoarthrodial joint that is unique in having fibrocartilaginous load-bearing surfaces and two sides that function in coordination [1]. This distinctive anatomy, combined with the high and repetitive stresses generated during mastication, parafunction, and speech, makes the joint susceptible to degenerative changes. Temporomandibular disorders (TMDs) comprise a heterogeneous group of musculoskeletal and neuromuscular disorders affecting the TMJ, masticatory muscles, and associated structures and are recognized as a common cause of chronic orofacial pain of non-dental origin [2]. Approximately 50%-80% of the general population may exhibit at least one sign of TMD during their lifetime, although a smaller proportion, approximately 20%-40%, experience symptoms requiring clinical care [3,4]. The etiology of TMD is multifactorial and includes biomechanical, traumatic, hormonal, genetic, and psychosocial factors [4].

Osteoarthritis (OA), a degenerative joint disease of the TMJ, is characterized by progressive alterations of the articular tissues accompanied by osseous changes involving the mandibular condyle and/or articular eminence [5]. Within contemporary diagnostic classifications, degenerative joint disease associated with arthralgia is classified as OA, whereas degenerative joint disease without arthralgia is classified as osteoarthrosis [6]. However, osseous findings may overlap with physiological remodeling and age-related changes, and isolated radiographic abnormalities do not necessarily indicate symptomatic disease; therefore, accurate imaging and standardized interpretation are essential [6]. TMJ pathologies and disorders also have important clinical implications during routine dental and orthodontic management [7]. Furthermore, the relationship between orthodontic interventions and the emergence of TMJ alterations in younger patient cohorts warrants close evaluation [8].

Magnetic resonance imaging (MRI) is the preferred imaging modality for evaluating the articular disc and other soft-tissue components of the TMJ, whereas computed tomography (CT) and cone-beam computed tomography (CBCT) are particularly useful for assessing osseous structures [9]. CBCT is increasingly used for TMJ assessment because it provides three-dimensional visualization of osseous structures with minimal superimposition and high spatial resolution, while generally delivering a lower radiation dose than multidetector CT; radiation exposure, however, varies according to the device, field of view, voxel size, and acquisition parameters, and cost is dependent on the clinical setting [10]. Low-dose tomographic protocols have also demonstrated high diagnostic validity for assessing three-dimensional condylar and TMJ morphology while minimizing radiation burden [11]. These characteristics make CBCT particularly useful for evaluating radiographic features of TMJ OA, including flattening, subchondral sclerosis, erosion, osteophyte formation, and subchondral cysts.

Importantly, such osseous changes are not restricted to symptomatic TMD populations and may also occur in asymptomatic and non-TMD individuals [12,13]. Furthermore, occlusal discrepancies and malocclusion traits have demonstrated distinct associations with the prevalence and distribution of specific TMJ signs and symptoms among young adults [14]. The presence of isolated flattening or localized sclerosis may reflect adaptive remodeling or normal variation rather than pathological OA, emphasizing the need to interpret CBCT findings in conjunction with clinical diagnostic criteria.

This review aims to: (1) summarize the range of osteoarthritic osseous changes detected on CBCT; (2) compare the prevalence and distribution of these changes between TMD and non-TMD populations; (3) evaluate the influence of demographic variables, including age and sex; (4) discuss the advantages and limitations of CBCT relative to other imaging modalities; and (5) examine current controversies and emerging directions in CBCT-based assessment of TMJ osteoarthritic changes.

## Review

Pathophysiology of TMJ OA

It is best to think of the development of TMJ OA as an active process, rather than just one of mechanical "wear and tear." The deterioration of the articular soft-tissue cover is the first stage in degeneration, followed by exposure and remodeling of the underlying subchondral bone [11]. The disease was long believed to be an inevitable consequence of aging, but it is now known that there is a significant inflammatory component, with cytokines and other inflammatory mediators generated by cartilage, bone, and synovium that promote matrix degradation and disruption of bone turnover [12]. For the TMJ, the relationship with chronological aging seems to be less strong than in some large weight-bearing joints, and local biomechanical overload and inflammatory signaling are particularly important.

The osseous consequences of this process are the features that CBCT is able to capture. Osteophyte formation and apposition of bone are reparative or adaptive processes, whereas condylar resorption is a loss of bone. Longitudinal three-dimensional studies have shown that there is a dynamic remodeling process within the osteoarthritic condyle, with both resorption and apposition occurring over time, with a static image representing a single point in a remodeling process [13].

Diagnostic criteria and the classification of TMJ OA

To compare imaging findings within and between populations, standardized diagnostic frameworks are key. The Research Diagnostic Criteria for Temporomandibular Disorders (RDC/TMD) was introduced in 1992, and the system included a dual-axis structure (Axis I addressed physical diagnosis, while Axis II addressed pain-related disability and psychosocial status) [14]. Each modality of the RDC/TMD Validation Project developed comprehensive image-analysis criteria; interexaminer reliability was lowest for panoramic radiography, intermediate for MRI, and highest for CT, which was used as the reference standard [14]. Based on these findings, cross-sectional tomographic imaging was concluded to be significantly better than two-dimensional radiography for detecting osseous OA. The criteria were revised and published in 2014 as the Diagnostic Criteria for Temporomandibular Disorders (DC/TMD), which was based on the original RDC/TMD but added updated and evidence-based algorithms without abandoning the dual-axis structure [15]. In DC/TMD, the osseous changes of DJD are characterized by the presence of either a subchondral cyst, erosion, generalized sclerosis, or osteophyte seen on CT or CBCT, whereas flattening and/or cortical sclerosis alone are considered indeterminate. In particular, the criteria recognize that imaging does not always reveal osseous changes in early disease and that a clinical diagnosis of DJD made on the basis of crepitus alone is not very sensitive or specific in the absence of imaging [16]. Therefore, CBCT is recommended when evaluating degenerative disease.

CBCT as an imaging modality for the TMJ

CBCT produces a volumetric data set obtained by a single rotation of a cone-beam X-ray beam, which can be reconstructed in various planes, and images of the bone compartment can be constructed without the superimposition or geometric distortion that can occur with panoramic and conventional tomography [8]. For the TMJ, this allows for separate analysis of the condyle, fossa, and articular eminence in corrected sagittal and coronal planes to better identify subtle changes in the cortex [8]. The main advantages of CBCT with respect to osseous TMJ assessment are its high spatial resolution, which is attributable to its small isotropic voxels, its lower radiation dose compared with multidetector CT, its comparatively low cost, and its growing availability in dental and maxillofacial practice [8,16]. In a systematic review and meta-analysis of the accuracy of CBCT for the detection of TMJ osseous defects, a pooled sensitivity of around 0.67 and pooled specificity of around 0.87 were reported, with a large area under the receiver operating characteristic curve (ROC) of about 0.84, and voxel size in the ranges studied did not significantly change the accuracy [17]. This profile of moderate sensitivity and high specificity suggests that CBCT is more likely to confirm the diagnosis of osseous change than to rule it out. There are also limitations. In patients with internal derangements, CBCT provides limited soft-tissue contrast and thus cannot assess the articular disc, retrodiscal tissues, joint effusion, or synovial inflammation, which are all key to the diagnosis of internal derangements [18]. As a result, MRI is complementary and is the imaging modality of choice when evaluating disc position or soft-tissue pathology; comparative studies have shown that CBCT is better for bony changes (hyperplasia, sclerosis, and condylar deformation), whereas MRI is better for disc displacement and effusion [19]. Furthermore, there is observer variability in the interpretation of subtle osseous changes and low interobserver correlation in borderline cases, so a diagnosis of OA should be made based on clear and unequivocal abnormalities. Finally, although X-ray doses are low, radiation exposure remains a concern, and low-dose CBCT protocols have demonstrated that adequate image quality of the TMJ can be obtained with a large reduction in the default dose [20].

The spectrum of osteoarthritic changes on CBCT

The osseous characteristics of TMJ OA found on CBCT are well known and are similar to those specified in the image analysis criteria of the RDC/TMD and DC/TMD. Flattening is a loss of the normal rounded convex contour of an articular surface, giving it a flat profile. It is the most commonly reported change in many general and clinical cohorts, but it is not very specific, and there is significant overlap between it and adaptive remodeling [21]. Subchondral (subcortical) sclerosis is characterized by radiopacity and thickening of the cortical or subcortical bone due to reactive bone deposition as a result of altered loading. Erosion is generally considered a more specific indicator of ongoing degeneration, as it is characterized by a loss of continuity of the outline of the cortex with an area of decreased bone density adjacent to the erosion. A reparative response is an osteophyte, which is a marginal bony outgrowth with sclerotic borders at the periphery of the articular surface, a characteristic of established disease. Subchondral cysts (also called pseudocysts or Ely cysts) are well-defined radiolucent spaces under the articular surface and are the least common but most diagnostic characteristic of DJD. The presence of at least one of a subchondral cyst, erosion, generalized sclerosis, or osteophyte is considered to reinforce the diagnosis of DJD based on the radiographic findings under the DC/TMD framework, while isolated flattening or localized sclerosis is regarded as indeterminate [22].

Changes in TMD populations - prevalence and distribution

CBCT imaging of referred TMD patients consistently shows a high amount of osseous change, but often with different proportions of individuals being affected by each specific feature, depending on age, diagnostic criteria, and referral pattern. In a retrospective study of 440 joints in 220 consecutive TMD patients attending a clinic, the most common condylar changes were flattening, surface erosion, and sclerosis, followed by deviation in form; osteophytes and subchondral cysts were observed in a smaller proportion of joints, while hypoplastic condyles were found in a minority [23]. In the 400 joints of a cross-sectional CBCT study of 200 TMD patients, osteophytes were the feature most commonly seen, followed by flattening, erosion, ankylosis, and sclerosis, which shows how the rankings of features can vary from sample to sample [24].

A recurring finding is that the nature of the changes varies with age in TMD and mixed cohorts, with proliferative and reparative features (e.g., osteophytes) becoming more common in older age groups, reflecting the shift from an early period of active erosive disease to later reparative disease [25]. Three-dimensional surface models have also been used in studies to demonstrate measurable differences between the morphology of osteoarthritic condyles and asymptomatic condyles, thus quantifying the changes detected on CBCT and demonstrating that they are not incidental but true differences in morphology between the two conditions [26].

The main challenge for diagnosis is that people who do not have any symptoms of TMD can have the same osseous changes as people who do. CBCT scans of an older, clinically asymptomatic, postmenopausal, edentulous woman revealed that flattening and sclerosis were more prevalent, whereas erosions were less prevalent, osteophytes were almost absent, and there was no correlation between the bony changes and whole-body BMD. The prevalence of TMJ changes was observed in a hand-OA cohort irrespective of TMJ symptoms, and the main pattern of changes was bone-productive (flattening, osteophytes, and subchondral sclerosis), corresponding to a late-stage, reparative radiographic picture, with the limitation of low observer agreement on subtle changes. Likewise, osseous change was observed in most adults 65 years and older who were imaged for any purpose, particularly in the form of subchondral cysts and osteophytes, and was more common in females [27].

Osseous change does not occur only in the elderly. In a consecutive sample of young adults, degenerative osseous TMJ changes were not uncommon even in those without a TMD diagnosis, with condylar erosion, flattening, and osteophyte formation present in significant numbers, and orthodontic factors were shown to be associated with the imaging changes, suggesting that nonpathological and iatrogenic influences also play a role in the imaging picture [28]. Together, these observations highlight that the "any osseous change" sign is too sensitive to be used as an isolated diagnostic criterion for OA and that the clinical context is critical.

A comparison between TMD and non-TMD individuals

When studies directly compare people with TMD and healthy controls, the differences and similarities are most striking, and the general consensus is that osteoarthritic changes occur more frequently and are more severe in people with TMD than in those who do not have TMD; however, there is significant overlap in the prevalence of osteoarthritic changes. A cross-sectional CBCT study of 60 patients with clinical TMD symptoms compared with 60 healthy patients showed that the TMD group had a higher incidence of degenerative bone changes, such as osteophytes, flattening, sclerosis, erosion, and pseudocysts, which were more severe in the TMD group [29]. In adolescent and young-adult populations, approximately a quarter to two-fifths of joints in patients with TMD have been reported to show osteoarthritic changes in controlled comparisons with asymptomatic or pre-orthodontic controls, compared with approximately a tenth of joints in controls [30]. For instance, symptomatic adolescents have been found to have radiographic OA in significantly greater proportions of their joints than same-age controls who underwent examination for tooth impaction or orthodontic assessment, and pre-orthodontic adolescents with malocclusion but without TMD have exhibited osseous changes at significantly lower frequencies than those with TMD.

The comparative literature that emerges is as follows: First, the direction of the difference is the same: having TMD is correlated with a higher amount of osseous change. Second, erosive and cystic (destructive) features discriminate between groups better than flattening or isolated sclerosis, as reflected in DC/TMD, which treats the latter as indeterminate [31]. Third, measurable osseous changes occur in a significant proportion of non-TMD individuals, limiting the specificity of any given change and making imaging a complement to a validated clinical assessment, not a replacement [32].

Limitations

A few methodological issues temper the interpretation of the comparative literature. Most investigations are retrospective and cross-sectional in nature, documenting a single time point in a dynamic remodeling process and making it impossible to make inferences about causation and/or progression. There are differences in case definitions, control selection, and thresholds used for osseous features to define "OA" between studies, which also influence detection rates (e.g., flattening is a low-specificity feature that is not consistently detected across studies) and thus the ability to pool the data. Also, acquisition parameters, voxel size, and reconstruction algorithms are not standardized, and although voxel size within the range of typical values does not appear to have a strong impact on accuracy, protocol variations are potential sources of variance. Interobserver agreement for subtle findings is often only moderately good, and the reporting of unilateral versus bilateral involvement is often asymmetric, making cross-study comparisons somewhat difficult. Last but not least, because CBCT cannot evaluate the disc or soft tissues, studies conducted only with CBCT always provide a limited picture of the joint and may misdiagnose soft-tissue pathology by attributing symptoms to osseous pathology.

Future directions

Three movements will help to reinforce the field. First, there are three-dimensional and longitudinal methods, such as surface-based shape correspondence and voxel-based superimposition, both of which can be used to quantify resorption and apposition over time rather than simply categorically scoring for presence or absence, which will provide greater sensitivity to early or progressive disease. Second, optimization of doses (such as validated low-dose protocols for CBCT) will help maintain the quality of diagnosis with minimal exposure, especially in children and for serial monitoring. Third, the development of artificial intelligence and machine-learning models to classify osteoarthritic changes on TMJ radiographs and CBCT is underway, which will aim to standardize interpretation and reduce intra- and interobserver variability in subtle-finding assessment, which is currently a problem. In addition to these technical advances, a more consistent application of the criteria for the detection of DC/TMD across studies would help make studies more comparable and facilitate more powerful meta-analysis.

## Conclusions

CBCT provides valuable three-dimensional visualization of osseous changes associated with TMJ OA. However, the presence of osseous changes alone should not be considered sufficient to establish a diagnosis of OA. CBCT findings should be interpreted in conjunction with a structured clinical assessment and relevant patient symptoms. Standardized image-analysis protocols, quantitative assessment, low-dose imaging approaches, and artificial intelligence-assisted interpretation may further improve the consistency and clinical utility of CBCT for evaluating TMJ osseous changes.
